# The insulinogenic effect of whey protein is partially mediated by a direct effect of amino acids and GIP on β-cells

**DOI:** 10.1186/1743-7075-9-48

**Published:** 2012-05-30

**Authors:** Albert Salehi, Ulrika Gunnerud, Sarheed J Muhammed, Elin Östman, Jens J Holst, Inger Björck, Patrik Rorsman

**Affiliations:** 1 Lund University Diabetes Centre, Clinical Research Centre, University Hospital, Lund University, Lund, Sweden; 2 Department of Applied Nutrition and Food Chemistry, Lund University, Lund, Sweden; 3 Department of Medical Physiology, The Panum Institute, University of Copenhagen, Copenhagen, Denmark; 4 Oxford Centre for Diabetes, Endocrinology and Metabolism, Oxford University, Oxford, OX3 7LJ, UK; 5 Applied Nutrition and Food Chemistry, Lund University, P.O. Box 124, 221 00 Lund, Sweden

**Keywords:** Amino acids, GIP-antagonist, Incretins, Insulin release, *In vitro*, Isolated Langerhans islets, Whey

## Abstract

**Background:**

Whey protein increases postprandial serum insulin levels. This has been associated with increased serum levels of leucine, isoleucine, valine, lysine, threonine and the incretin hormone glucose-dependent insulinotropic polypeptide (GIP). We have examined the effects of these putative mediators of whey’s action on insulin secretion from isolated mouse Langerhans islets.

**Methods:**

Mouse pancreatic islets were incubated with serum drawn from healthy individuals after ingestion of carbohydrate equivalent meals of whey protein (whey serum), or white wheat bread (control serum). In addition the effect of individual amino acid combinations on insulin secretion was also tested. Furthermore, the stimulatory effects of whey serum on insulin secretion was tested *in vitro* in the absence and presence of a GIP receptor antagonist ((Pro(3))GIP[mPEG]).

**Results:**

Postprandial amino acids, glucose-dependent insulinotropic polypeptide (GIP) and glucagon-like peptide 1 (GLP-1) responses were higher after whey compared to white wheat bread. A stimulatory effect on insulin release from isolated islets was observed with serum after whey obtained at 15 min (+87%, *P* < 0.05) and 30 min (+139%, *P* < 0.05) postprandially, compared with control serum. The combination of isoleucine, leucine, valine, lysine and threonine exerted strong stimulatory effect on insulin secretion (+270%, *P* < 0.05), which was further augmented by GIP (+558% compared to that produced by glucose, *P* < 0.05). The stimulatory action of whey on insulin secretion was reduced by the GIP-receptor antagonist (Pro(3))GIP[mPEG]) at both 15 and 30 min (−56% and −59%, *P* < 0.05).

**Conclusions:**

Compared with white wheat bread meal, whey causes an increase of postprandial insulin, plasma amino acids, GIP and GLP-1 responses. The *in vitro* data suggest that whey protein exerts its insulinogenic effect by preferential elevation of the plasma concentrations of certain amino acids, GIP and GLP-1.

## Background

Dairy products produce higher insulin responses (Insulin index, II, 90–98) than expected from their comparatively low glycemic indices (GI 15–30) [1,2]. Insulinogenic effects from dairy products have been observed in healthy subjects, both when ingested as a single meal [1], and when included into a mixed meal [2,3]. The insulin-releasing capacity of dairy products has been attributed to the protein fraction, and both whey and casein have been shown to stimulate insulin secretion in healthy subjects [4,5]. Particularly the whey fraction and release of amino acids during digestion has been proposed to underlie the insulinogenic properties of milk [6]. Indeed, several amino acids are known to stimulate insulin secretion both *in vivo* and *in vitro*[7-13]. Previous work has shown that postprandial plasma concentrations of isoleucine, leucine, valine, lysine and threonine are elevated following a whey meal [4], and these amino acids have been implicated as mediators of the insulinogenic effect [6]. In addition, the incretin hormone GIP has been shown to be present at higher postprandial levels after ingestion of whey compared to white wheat bread (WWB) [4,6,14], and may thus contribute to the insulinotropic effect.

The objective of this study was to investigate the mechanisms underlying the insulinogenic effect of whey protein. We aimed to determine whether or not serum responses of amino acids (isoleucine, leucine, valine, lysine and threonine) and GIP, following whey ingestion exerted their effects on insulin stimulation directly on the pancreatic β-cells. We have compared the effects of postprandial serum withdrawn from healthy individuals after ingestion of equi-carbohydrate portions of whey or white wheat bread (WWB), respectively, on insulin secretion *in vitro.* The impact of isoleucine, leucine, valine, lysine and threonine, as well as of a mixture thereof was studied using an *in vitro* model with isolated islets from mice [6]. In the light of our previous studies showing significant increases in serum GIP following oral administration of whey, *in vitro* insulin release in response to selected amino acids was tested with or without addition of a GIP antagonist [6].

## Materials and methods

### Human meal studies

#### Serum for *in vitro* analysis of insulin secretion of Langerhans islets

Six (4 M, 2 W), non-smoking healthy volunteers, aged 20–30 y, with normal fasting blood glucose and body mass indices and without any history of previous drug treatment participated in the study. Two meals, whey protein and white wheat bread (WWB), matched with regard to carbohydrate content (25 g) were provided in the morning after an overnight fast. Lactose was the carbohydrate source in the whey meal, and starch in the WWB meal. The protein amount was 3.7 g in the WWB meal and 16.7 g in the whey meal. The participants were instructed to avoid alcohol, excessive physical activity and food rich in dietary fibers on the day before the test and to eat a standardized meal consisting of white wheat bread between 9 and 10 pm and then nothing except small amounts of water. When reporting at the laboratory in the morning, a peripheral catheter was inserted into an antecubital vein and fasting blood was sampled (time 0). Thereafter, the test meal was served and completed within 12 min. Coffee or tea (150 ml) was served 15 min after start, and each subject maintained the same drink throughout the study. Blood samples were then taken 7.5, 15, 30 and 45 min after the start of the meal. Sera obtained after whey ingestion (“whey serum”) and WWB (“control serum”) were stored at -20°C until analyzed. Blood samples were also taken at 0, 15, 30 and 45 min for analysis of free amino acids, GIP and GLP-1 in plasma. All test subjects gave their informed consent and were aware of the possibility of withdrawing from the study at any time. The study was approved by Ethics Committee of the Faculty of Medicine at Lund University.

#### Serum for *in vitro* analysis of insulin secretion of Langerhans islets with or without GIP receptor antagonist ((Pro(3))GIP[mPEG])

The meal study was performed in the same way as mentioned above, with the difference that it was 12 healthy test subjects (7 W, 5 M). One test drink and one reference drink were included in the study, which were served as breakfast on two different occasions. The test drinks consisted of 18 g of whey protein. The reference drink was glucose in 250 g water. Blood samples were taken at 0, 15 and 30 min.

### Blood analysis

Free amino acids were purified by mixing 200 μl 10% sulfosalicylic acid with 800 μl plasma to precipitate high molecular-weight proteins. The amino acids were then analyzed with an amino acid analyzer (LC 5001; Biotronik, München, Germany) by ion-exchange chromatography. The amino acids were separated by using standard lithium citrate buffers of pH 2.85, 2.89, 3.20, 4.02 and 3.49. The post column derivatization was performed with ninhydrin [15].

Plasma glucose-dependent insulinotropic polypeptide (GIP) and glucagon-like peptide 1 (GLP-1) concentrations were measured after extraction of plasma with 70% ethanol. For the GIP radioimmunoassay [16], antiserum R65 (directed against the C-terminus) was used. Human GIP and ^125^I-labelled human GIP (70 Bq/nmol) were used for standards and tracer. The plasma concentrations of GLP-1 were measured as previously reported [17] using antibodies directed against the amidated C-terminus of GLP-1 (code no. 89390) against standards of synthetic GLP-1 7–36 amide. For both assays, sensitivity was < 1 pmol/l, the intra-assay CV was < 6% at 20 pmol/l, and the recovery of standard (which was added to plasma before extraction) was ≈ 100% when corrected for losses inherent in the plasma extraction procedure.

### *In vitro* analysis of Langerhans islets

#### Animals

Female mice of the NMRI strain (B&K Universal, Sollentuna, Sweden), weighing 25–30 g, were used for all experiments. The mice were fed a standard pellet diet (B&K Universal, Sollentuna, Sweden) and tap water *ad libitum*. The mice were killed by cervical dislocation followed by decapitation (with ethical approval).

#### Drugs and chemicals

Collagenase (CLS 4) was obtained from Sigma Chemicals Corp. (St. Louis, MO., USA). Bovine serum albumin was from ICN Biomedicals (High Wycombe, England). GIP-antagonist ((Pro(3))GIP[mPEG]) was kindly provided by Professor PR Flatt, (University of Ulster, Coleraine, UK). All other chemicals were from Merck AG (Darmstadt, Germany). The radioimmunoassay kits for insulin and glucagon were from Diagnostika (Falkenberg, Sweden) and Eurodiagnostika (Malmö, Sweden), respectively.

#### Experimental protocol

All islet experiments were performed with mouse pancreatic islets isolated by retrograde injection of a collagenase solution *via* the bile-pancreatic duct [18-20]. The islets were collected under a stereomicroscope at room temperature, and transferred to incubation vials (12 islets per 1.0 ml incubation buffer in each vial). They were then pre-incubated for 30 min at 37°C in 1 ml of Krebs-Ringer bicarbonate (KRB) buffer, pH 7.4, supplemented with 10 mmol/l HEPES and 0.1% bovine serum albumin and 1 mmol/l glucose as previously described [18]. Each batch of islets was then gassed with 95% O_2_ and 5% CO_2_ to obtain constant pH and oxygenation. After pre-incubation, the buffer was changed to an identical medium in the presence or absence of different test agents (human serum, amino acids and/or GIP) and 8.3 mmol/l glucose. The KRB buffer was compensated for the glucose levels in the serum (measured separately) when the serum was added to the buffer to a final concentration of 8.3 mmol/l. Islets were thereafter incubated for 60 min at 37°C in a shaking incubator (30 cycles per min). Immediately after incubation, aliquots of the medium were removed for radioimmunoassay of insulin [21]. All experiments were performed in at least four replicates.

Serum from 6 healthy subjects was pooled yielding one sample for each time point and product (see human meal study above). Thereafter, the glucose, insulin, GIP and GLP-1 concentrations, as well as amino acid levels were analyzed in postprandial whey and control serum. The insulin concentrations present originally in the serum were subtracted from the concentrations obtained after the incubation with isolated islets in order to determine the amount of insulin secreted during the incubation.

Previous work has established that the plasma concentrations of isoleucine, leucine, valine, lysine and threonine are elevated following a whey meal [4]. The impact of each of these amino acids at a standard concentration of 4 mM on insulin secretion from isolated islets was therefore examined. In addition, the effects of a cocktail consisting of 4 mM of each amino acid with and without 100 nM GIP were tested. These are standard concentrations used for *in vitro* work [22,23]. Furthermore, the stimulatory effects of whey serum (obtained at 0, 15 and 30 min after meal ingestion) on insulin secretion was tested *in vitro* in the absence and presence of a 100 nM GIP receptor antagonist ((Pro(3))GIP[mPEG]) using pooled serum from 12 healthy subjects (see human meal study, above).

#### Statistical methods

The results are expressed as means ± SEM for the indicated number of observations. Probability levels of random differences were determined by analysis of variance followed by Tukey-Kramer’s multiple comparisons test using GraphPad Prism^TM^, (version 4.03; GraphPad Software Inc, San Diego, CA), when comparing the differences in insulin releasing capacities of whey and control serum, and to evaluate the effect of the GIP antagonist. Significant differences in insulin release among different amino acids were assessed by ANOVA followed by Tukey’s multiple comparisons test (MINITAB, release 14.13; Minitab Inc, State Collage, PA). Differences resulting in *P* < 0.05 were considered statistically significant.

## Results

### Post-prandial plasma response of amino acids, GIP and GLP-1

**Figure 1:**
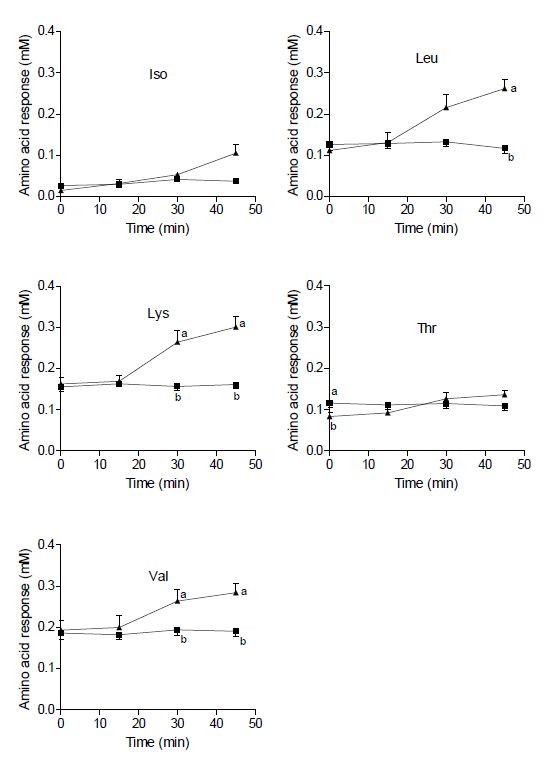
**Amino acid responses in humans.** Mean (± SEM) values of isoleucine, leucine, valine, lysine and threonine in plasma obtained at different time points in response to the whey (▴) and WWB (■) meal.

**Figure 2:**
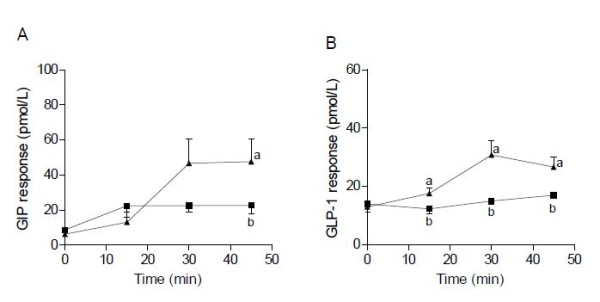
**Incretin responses in humans.** Mean (± SEM) values of GIP **(A)** and GLP-1 **(B)** in plasma obtained at different time points in response to the whey (▲) and WWB (■) meal.

The mean plasma concentration of valine, isoleucine, leucine, threonine and lysine determined 0, 15, 30 and 45 min after ingestion of whey and WWB, respectively, are presented in Figure 1. At 45 min, the concentrations were 2.2-, 2.8-, 1.5-, 1.2- and 1.9-fold higher after ingestion of whey than WWB for leucine, isoleucine, valine, threonine and lysine, respectively. The plasma levels of both GIP and GLP-1 were ~2-fold higher at 30 and 45 min after ingestion of whey compared with WWB (Figure 2).

### Effects of post-prandial serum on *in vitro* insulin secretion

**Figure 3:**
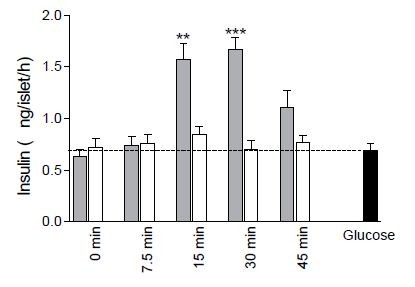
**Insulin secretion of Langerhans islets incubated with human serum.** Insulin secretion of isolated pancreatic islets incubated with serum obtained from healthy subjects after ingestion of whey (grey bars) and WWB (control, white bars), and 8.3 mM glucose alone (reference, black bar). The islets were pooled from 8 different NMRI mice and values are means ± SEM for 10 observations in each group performed at three different occasions. Significant differences between whey and control serum for each time point are denoted by **P < 0.01, ***P < 0.001.

We examined the effects of whey (grey bars) or control (white bars) serum samples obtained at 0, 7.5, 15, 30 and 45 min after ingestion of the meal on insulin secretion from freshly isolated mouse islets exposed to 8.3 mM glucose (Figure 3). Whereas control serum was without effect on insulin secretion, whey serum obtained at 15 and 30 min after meal ingestion stimulated insulin secretion >2.5-fold.

### Effects of amino acids on *in vitro* insulin secretion

**Figure 4:**
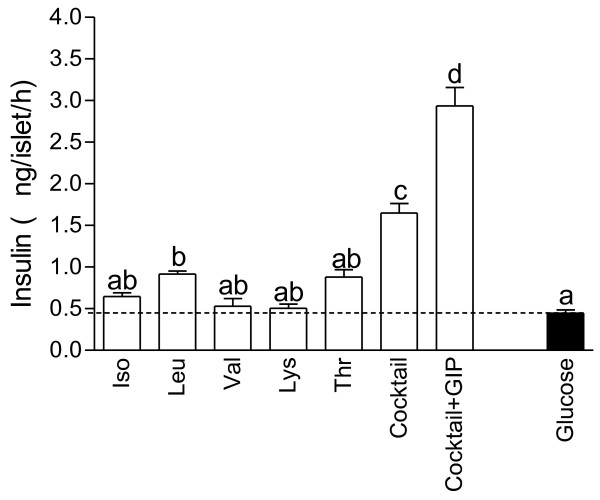
**Insulin secretion of Langerhans islets incubated with amino acid.** Insulin secretion from isolated pancreatic islets incubated at 8.3 mM glucose in the absence or presence of leucine, isoleucine, valine, lysine and threonine, a cocktail of the 5 amino acids or a cocktail + GIP. Values are means ± SEM for 8 observation in each group performed at three different occasions. The experiments were performed on the islets isolated and pooled from 6 different NMRI mice. Values with different letters are significantly different, *P* < 0.05.

We tested the effects of the five amino acids that are increased in response to whey (leucine, isoleucine, valine, threonine and lysine; cf. Figure 1). When tested at a concentration of 4 mM, only leucine (+105%, *P* < 0.05) evoked a statistically significant stimulation of insulin secretion relative that produced by glucose alone (Figure 4). A cocktail consisting of all five amino acids (each included at a concentration of 4 mM) increased insulin release beyond (+270%, *P* < 0.05) that obtained with glucose alone or any of the amino acids when they were tested individually. The effect of the cocktail was enhanced ~2-fold (*P* < 0.05) by GIP.

### Effects of a GIP antagonist on insulin secretion

**Figure 5:**
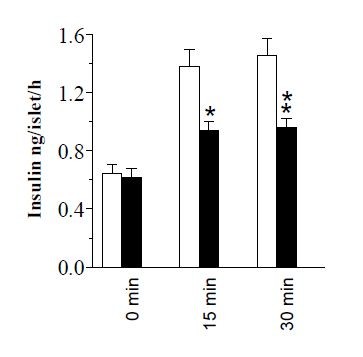
**Insulin secretion of Langerhans islet incubated with human serum in presence of a GIP-receptor antagonist.** Insulin secretion from isolated mice pancreatic islets incubated at 8.3 mmol/l glucose + whey serum (obtained before intake time 0 as well as at 15 and 30 min after meal ingestion) in the absence (white bars) and presence (black bars) of GIP-receptor antagonist ((Pro(3))GIP[mPEG]) (100 nmol/l). Values are means ± SEM for 8 observations in each group performed at three different occasions on the isolated islets from 6 different NMRI mice. Significant differences between absence and presence of the antagonist are denoted by * *P* < 0.05; * * *P* < 0.005.

The stimulatory action of whey serum on insulin secretion, with or without added GIP-receptor antagonist, is seen in Figure 5. The insulin secretion, exceeding that produced by glucose alone, was significantly reduced in the presence of the GIP-receptor antagonist (100 nmol/l) with −56% at 15 min (*P* < 0.05) and −59% at 30 min (*P* < 0.01).

## Discussion

In this study whey ingestion resulted in a 1.2- to 2.8-fold increase in plasma concentration of leucine, isoleucine, valine, threonine and lysine relative the basal plasma levels and those obtained after ingestion of white wheat bread. It has previously been described that specific mixtures of amino acids do stimulate insulin secretion [6,10]. Thus, it is conceivable that the observed stimulation of insulin secretion is due to an increased level of the specific amino acids *in vivo*. The amino acid cocktail was shown to be superior to the single amino acids, with respect to *in vitro* insulin secretion, in support of such an opinion. Additionally, adding GIP to the amino acid cocktail further enhanced insulin stimulatory effect on the β-cells, indicating an involvement also of this incretin.

GIP as well as GLP-1 is a strong insulinotropic agent [24], and it has recently been reported that the two incretins have different effects on the insulin secretion, with GIP appearing to be more effective at normoglycemic levels and GLP-1 during hyperglycemia [25]. In previous studies we have demonstrated that whey protein affects GIP rather than GLP-1 [4,6,14]. Other studies have reported stimulation of GLP-1 as well [26] and in the present study whey protein clearly stimulated both GIP and GLP-1. Stimulation also of GLP-1 provides an alternative explanation for the insulinogenic action of whey. In the present study it was evident that whey ingestion increased the plasma concentration of incretins more than white wheat bread in healthy humans. Further, addition of a GIP receptor antagonist decreased the *in vitro* insulinotropic action of whey serum significantly, and future work should address the mechanism by which whey enhances release of GIP from the enteric K-cells.

Insulin secretion in response to amino acids is interesting in terms of treatment of type-2 diabetes. Most of the insulinogenic amino acids trigger insulin secretion by mechanisms that differ from those utilized by glucose [27]. In type-2 diabetes, the insulinogenic effects of amino acids may remain unaffected even after long-term diabetes [12,14,28]. Interestingly, addition of essential amino acids to a diet in poorly regulated elderly patients with type 2 diabetes improved metabolic control and lowered fasting blood glucose and serum insulin levels, as well as lowered levels of glycated hemoglobin (HbA1c) [29,30]. Possibly, the improved metabolic control results from increased muscle mass and improved insulin sensitivity, in addition to the insulinotropic effect of the amino acid supplementation.

A recent study identified the whey fraction, rather than the casein fraction, as the major insulin secretagogue in milk [4]. The different insulin-stimulating capacity of the whey and casein fractions may be secondary to differences in the rate of gastric emptying and digestion, with whey proteins being classified as rapidly released proteins [31,32]. It should be noted, however, that “whey serum” (i.e. obtained from subjects that had ingested whey protein) exerts a direct stimulatory effect on insulin secretion from isolated mouse islets. We therefore conclude that whey increases the plasma concentration of metabolites with insulinotropic capacity that act directly on the β-cells. In this context it is noteworthy that the stimulatory effect of the whey serum was strongest at 15 and 30 min after ingestion and that no stimulation was seen at 45 min.

The stimulation of insulin release by the whey serum *in vitro* was not caused by any changes in glucose resulting from the addition of the serum samples, as this was adjusted for. Although several metabolic and hormonal parameters could be differentially modified in the serum from whey-ingested subjects, the observation that GIP was elevated may be of particular significance. Indeed, inclusion of the GIP-antagonist ((Pro(3))GIP[mPEG]) in the incubation media markedly suppressed the stimulatory effects of the serum from whey-ingested subjects on insulin secretion from isolated islets. However, it should be emphasized that the results of present investigation illustrate an *in vitro* observation which needs to be verified in an appropriate *in vivo* animal model. Moreover, it will be interesting to confirm these observations using human islets to further study the differences or similarities in this context.

In conclusion, our work on isolated mouse pancreatic islets suggests that the insulinogenic capacity of whey proteins is mediated by postprandial elevation of specific plasma amino acids, and GIP. In addition, plasma GLP-1 levels were elevated and this may exert an additional stimulatory effect and the relative influence of the mediators of insulin secretion remains to be established.

## Abbreviations

GI: Glycemic index; GIP: Glucose-dependent insulinotropic polypeptide; GLP-1: Glucagon-like peptide 1; II: Insulinemic index; WWB: White wheat bread.

## Competing interest

The authors declare that they have no competing interest.

## Authors’ contribution

AS was responsible for the *in vitro* studies and was involved in the evaluation and in writing the paper. UG coordinated the meal study and collected serum for the *in vitro* analysis and was involved in the evaluation and writing the paper. SJM was involved in the *in vitro* studies and in the evaluation. EÖ was involved in the study design and in the evaluation and in writing the paper. JJH was responsible for the incretin analysis and was involved in the evaluation. IB was responsible for securing the funding and was involved in the design and the evaluation and the writing of the paper. PR was involved in the design and the evaluation and writing of the paper. All authors read and approved the final manuscript.
